# Evaluation of mRNA Expression of *CD244* and Its Adapter Molecules in CD8^+^ T Cells in Acute Leukemia

**DOI:** 10.61186/ibj.3843

**Published:** 2023-06-27

**Authors:** Maryam Mohammadi, Hossein Asgarian-Omran, Ahmad Najafi, Reza Valadan, Hossein Karami, Mohammad Naderisoraki, Ehsan Zaboli, Mohammad Eslami, Mohsen Tehrani

**Affiliations:** 1 Department of Immunology, School of Medicine, Mazandaran University of Medical Sciences, Sari, Iran;; 2 Gastrointestinal Cancer Research Center, Mazandaran University of Medical Sciences, Sari, Iran;; 3 Molecular and Cell-Biology Research Center, Mazandaran University of Medical Sciences, Sari, Iran;; 4 Thalassemia Research Center (TRC), Hemoglobinopathy Institute, Mazandaran University of Medical Sciences, Sari, Mazandaran Iran;; 5 Department of Hematology and Oncology, Imam Khomeini hospital, Mazandaran university of Medical Sciences, Sari, Iran

**Keywords:** Acute myeloid leukemia, CD244, EAT-2

## Abstract

ABSTRACT

**Background:**

This study investigated the role of the ICR, *CD244*, and its adapter molecules, in CD8^+^ T cells in acute leukemia.

**Methods:**

Blood samples were obtained from 21 ALL and 6 AML patients and 20 control subjects. Relative gene expression of *CD244*, *SAP*, *EAT-2*, and LncRNA-GSTT1-AS1 were evaluated using qRT-PCR.

**Results:**

Expression of *CD244*, *SAP*, and *EAT-2* were significantly lower in CD8^+^ T cells from ALL patients than those from control subjects. Interestingly, the expression of *SAP* was much lower than that of *CD244*, indicating a lower ratio of *SAP* to *CD244*. Also, *SAP* expression was significantly lower in AML patients compared to the control group. Expression of LncRNA-GSTT1-AS1 showed no significant difference in ALL and AML patients compared to control subjects.

**Conclusion:**

The low *SAP*/*CD244* expression ratio in CD8^+^ T cells in ALL suggests an inhibitory role for *CD244* in ALL.

## INTRODUCTION

Acute leukemia, including ALL and AML, are two common leukemias throughout the world^[^^1^^,^^2^^]^. Treatments for leukemia include chemotherapy, radiation therapy, and hematopoietic stem cell transplantation, which have many side effects and limited efficacy^[^^1^^]^. One of the problems in chronic infections and cancers, which the immune system suffers, is the dysfunction of T cells, known as T-cell exhaustion^[^^3^^]^. Exhausted T cells show overexpression of some ICRs, which bind to their ligands on the surface of antigen-presenting and tumor cells. The most important ICRs are PD-1, Tim-3, LAG-3, TIGIT, and CD244. Blockade of these receptors, so-called immune checkpoint blockade, using monoclonal antibodies or other approaches, improved T cell function and slowed progression of the disease in animal models of cancer, as well as in human cancers^[^^4^^]^. In leukemia, however, the role of ICRs has not fully been investigated; thus, immune checkpoint blockade therapy in leukemias has not yet been approved by the US Food and Drug Administration (FDA). 

CD244, a member of the SLAM family, is expressed on the surface of several immune cells, including T-cells, NK cells, basophils, monocytes, dendritic cells, and myeloid-derived suppressor cells. Its ligand, CD48, is expressed on the surface of various hematopoietic cells^[^^5^^]^. Once CD244 binds to its ligand, its ITSM motifs binds to the adapter molecule, SAP, which leads to an activating signal. However, it can bind to the other adapter molecule, EAT-2, resulting in an activating or inhibitory signal^[^^6^^,^^7^^]^. It is supposed that expression levels, availability, and competitive binding of the adapter molecules, SAP and EAT-2, determine whether CD244 mediates an activating or inhibitory signal, as adequate concentrations of functional SAP leads to CD244 activating signals^[^^4^^]^. Therefore, the ratio of SAP to CD244 plays an important role in determining an activating or inhibitory role for this molecule^[^^4^^]^. CD244 has been shown to be overexpressed in the exhausted T cells in chronic infections, such as in hepatitis B^[^^8^^]^, as well as in some malignancies, including multiple myeloma^[^^9^^]^ and AML^[^^10^^]^. Of note, blockade of CD244, using monoclonal antibodies, increased the population of virus-specific T cells and expression of CD107a, perforin, IFN-, TNF-, and IL-6 in *in vitro* models^[^^4^^]^. Study of CD244 and its adapter molecules, SAP and EAT-2, in CD8^+^ T cells in acute leukemia would be helpful to further understand T cell exhaustion-related pathways and to find therapeutic targets for acute leukemia. Hence, this study aimed to investigate the expression of CD244 and its related adapter molecules in CD8^+^ T cells in acute leukemia^[^^6^^]^.

## MATERIALS AND METHODS

**Patients and controls**

The sample size was calculated based on previous studies^[^^11^^-^^13^^]^. Twenty-one ALL patients and six AML patients, referred to the Outpatient Clinic or the Hematology and Oncology Wards of Imam Khomeini Hospital and Bou-Ali Sina Hospital (both affiliated to Mazandaran University of Medical Sciences, Sari, Iran), were included in the study. Diagnosis of the disease was carried out via clinical evaluations, blood cell count, and morphology, along with the immunophenotyping of blood cells in the peripheral blood sample or the bone marrow, according to World Health Organization (WHO) criteria^[^^14^^]^. A questionnaire, including demographic, clinical and laboratory information, was completed separately for each study subject. Twenty healthy volunteers were also included in the study as a control group. Patients with chronic viral diseases, such as hepatitis B, hepatitis C and HIV, those with a history of autoimmune diseases or malignancies other than ALL and AML, or taking chemotherapy or immune-suppressive drugs, were excluded from the study. In total, 21 ALL (8 males and 13 females, mean age 15.94 years) and 6 AML (5 males and 1 female, mean age 45.66 years) patients, as well as 20 control subjects (9 males and 11 females, mean age 22.5 years) participated in the study. An amount of 8-10 mL of heparinized peripheral blood sample was taken from each study subject. 

**Isolation of CD8** ^+^ **T lymphocytes by magnetic-activated cell separation**

Heparinized peripheral blood samples were collected from patients and controls, and PBMCs were isolated by density gradient centrifugation on Lymphosep (Biosera, Nuaille, France). Isolated PBMCs were washed twice with RPMI-1640 culture medium (Biosera). Viability of the isolated cells was determined by trypan blue staining. CD8^+^ T cells were then purified from PBMCs using the magnetic-activated cell separation by means of CD8 microbeads (Miltenyi Biotec, Germany). To check the purity of the isolated CD8^+^ T cells, 2 10^5^ cells were stained with anti-CD8-FITC (Clone SK1, 0.125 µg/test, eBioscience, San Diego, US) and anti-CD3-PE (Clone UCHT1, 0.06 µg/test, eBioscience). Appropriate amounts of isotype-matched control antibodies were also used to subtract the background staining. Samples were then analyzed on a Partec PAS flow cytometer system (Partec GmBH, Munster, Germany) using the FlowMax software (version 2.82). 

**Quantitative reverse transcription polymerase chain reaction**

Total RNA was isolated from CD8^+^ T cells using the FavorPrep Blood/Cultured Cell Total RNA Mini Kit (*Favorgen,* Taiwan) based on the manufacturer’s protocol. The quantity and quality of the isolated RNA were confirmed by nano-spectrophotometer and electro-phoresis, respectively. The cDNA was synthesized using a cDNA synthesis kit (Yekta Tajhiz Azma, Tehran, Iran). qRT-PCR was performed using Real Q Plus 2× Master Mix (High Rox, Ampliqon, Denmark) reagent on an ABI Step one plus Real-Time system (Applied Biosystems, USA) with specific primers for *CD244*, *SAP*, *EAT-2*, and LncRNA-GSTT1-AS1, as well as *ACTB*, as a housekeeping gene. PCR was performed using 10 pmol of each forward and reverse primer, 1 µL of cDNA, 10 µL of Master mix, and 7 µL of PCR grade water and amplified at 95 °C for initial denaturation, followed by 40 cycles at 95 °C for 15 seconds, 60 °C for 30 seconds, and extension at 72 °C for 30 seconds. After normalizing to *ACTB*, relative expression levels of *CD244*, *SAP*, *EAT-2*, and LncRNA-GSTT1-AS1 was determined using the 2^-ΔΔCt^ method^[^^15^^]^.

**Statistical analysis**

Statistical analyses were performed using SPSS 18 software. Results are shown as mean ± SEM. Using Kolmogorov-Smirnov test, normal distribution of the studied parameters was checked, and parametric or non-parametric tests were used. P < 0.05 was considered statistically significant.

## RESULTS

**mRNA expression of** ***CD244*** **and its adapter molecules in patients and controls**

**Fig. 1:**
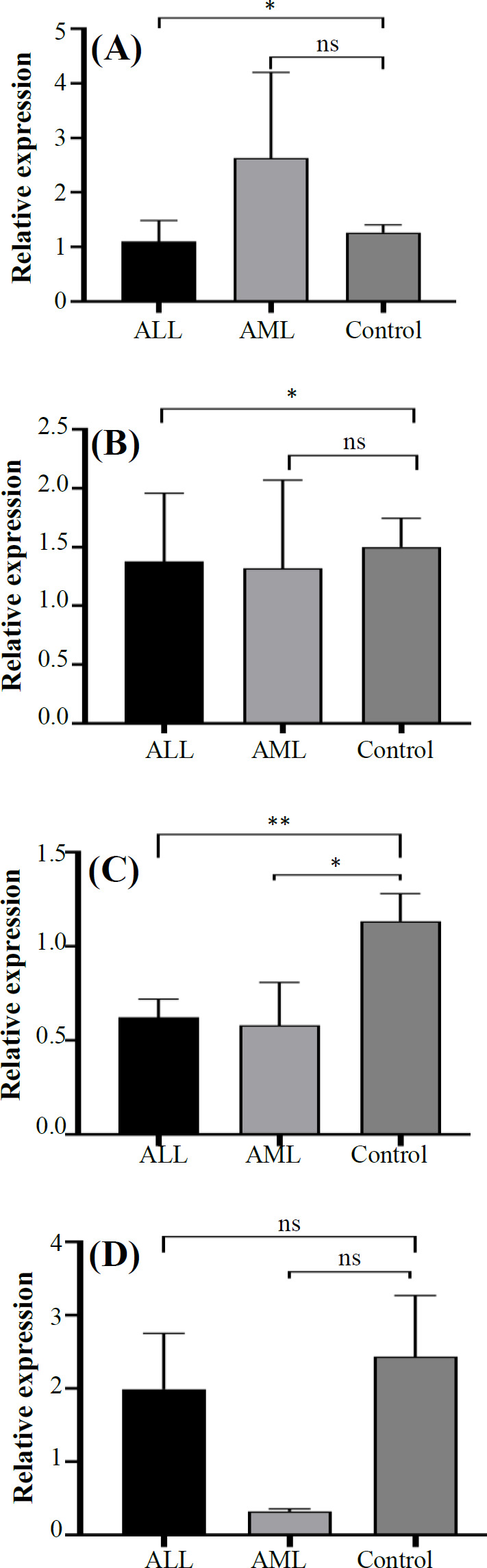
Relative mRNA expression of *CD244* (A), *EAT-2* (B), *SAP* (C), and LncRNA-GSTT1-AS-1 (Lnc-CD244; D) in ALL and AML patients and control subjects. The mRNA expression of *CD244*, *SAP*, *EAT-2*, and LncRNA-GSTT1-AS-1 was determined using qRT-PCR, normalized to that of *ACTB*, and expressed as relative expression. Vertical bars represent mean ± SEM (^*^p < 0.05; ^**^p <0.01)

Expression of *CD244* and *EAT-2* in patients with ALL was significantly lower than the control group (p = 0.0225 and p = 0.0375, respectively), while their expression was not significantly different between AML patients and the control group (p = 0.7785 and p = 0.5670; Fig. 1A and 1B). *SAP* expression in both ALL and AML patients was significantly lower than the control group (p = 0.0031 and p = 0.0155, Fig. 1C). 

**LncRNA-GSTT1-AS1 expression in patients and controls**

The relative mRNA expression of LncRNA-GSTT1-AS1, a long noncoding RNA, related to CD244 expression, known as LncRNA-CD244,^[16]^ was evaluated in CD8^+^ T cells from patients with ALL and AML, as well as the control group. The results of qRT-PCR showed that although its expression was lower in both ALL and AML patients than the control group, the differences were not statistically significant (p = 0.3273 and p = 0.5457, respectively; Fig. 1D).

**Correlations between** ***CD244*** **and** ***SAP*** **expression in ALL patients**

**Fig. 2:**
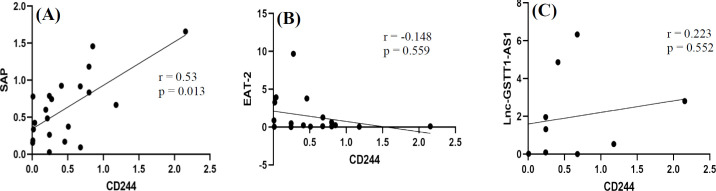
Correlations between mRNA expressions of *CD244* and its adapter molecules in ALL patients. Pearson’s correlation analyses between *CD244* and *SAP* (A), *CD244* and *EAT-2 *(B), and *CD244* and LncRNA-GSTT1-AS1 (C) gene expressions in ALL patients.

Correlation analysis of the expression level of *CD244* with that of its adapter molecules showed a direct correlation between *CD244* and *SAP* expression in ALL patients (r = 0.53; p = 0.013; Fig. 2A). However, the correlations between the expression level of *CD244* with that of *EAT-2* and LncRNA-GSTT1-AS1 were not statistically significant (Fig. 2B and 2C).

## DISCUSSION

CD244 is expressed on the surface of NK cells, T cells, dendritic cells, and other immune cells^[^^4^^]^. Basically, CD244 has been recognized as an ICR; however, its adapter molecules, including SAP and EAT-2, play important roles in determining whether CD244 produces an activating or inhibitory signal^[^^4^^]^. In this study, we focused on acute leukemia, including ALL and AML, and examined the relative mRNA expression of *CD244* and its downstream adapter molecules, *SAP* and *EAT-2*, in CD8^+^ T cells. 

Previously, it has been shown that *CD244* is overexpressed on exhausted CD8^+^ T cells in chronic infections, and its expression correlates with that of *PD-1*^[^^7^^,^^17^^]^. *CD244* also showed an overexpression on the exhausted CD8^+^ T cells in some human cancers, like melanoma^[^^18^^,^^19^^]^ and multiple myeloma^[^^9^^]^. In the present study, in ALL patients, *CD244* expression was significantly lower in CD8^+^ T cells than the control subjects, while it was not significantly different between AML patients and controls. Since these results were not in line with the previous studies, we then measured the relative expression of *SAP* and *EAT-2* in CD8^+^ T cells in ALL and AML patients. SAP binds to ITSM domainin the cytoplasmic part of CD244, which induces an activating signal. In the absence of sufficient concentrations of SAP, CD244 binds to phosphatases, and inhibitory signal transduction occurs. Alternatively, CD244 could bind to EAT-2, which is associated with activating or inhibitory signal transduction^[^^7^^]^. In our study, although both *CD244* and *SAP* showed reduced expression in CD8^+^ T cells in ALL patients, *SAP* expression was much lower than that of *CD244*. Also, expression of *CD244* and *SAP* were positively correlated with each other. As mentioned above, it has been shown that signaling function of CD244 depends on the concentration of its downstream adapter molecules. In the presence of adequate expression of SAP, it is more likely that this adapter molecule binds to the ITSM domain of the CD244 molecule, resulting in an activating signal; however, in the absence of enough expression of SAP, other adapter molecules in the downstream of CD244, such as phosphatases, bind to the CD244 tyrosine domain, leading to an inhibitory signal^[^^4^^]^. Since our results indicated that *SAP* expression in CD8^+^ T cells from ALL patients was much less than that of *CD244*, we assumed that the ratio of *CD244* to *SAP* expression was lower in CD8^+^ T-cells in ALL than in control subjects, thus the receptor would exhibit an inhibitory function.

In this study, we measured the expression level of the LncRNA-GSTT1-AS1 in CD8^+^ T cells from ALL and AML patients. LncRNAs are a recently discovered class of RNAs with a length of more than 200 base pairs that do not encode a protein^[^^20^^]^. Many LncRNAs have been identified in mammalian genomes that regulate the expression of genes related to T-cell differentiation^[^^21^^]^. A previous study also found out that in tuberculosis, as a chronic infection, the expression of *CD244* and then LncRNA-GSTT1-AS1, known as LncRNA-CD244, increased in CD8^+^ T cells, which led to decreased IFN- and TNF- production and impaired immune response to infection^[16]^. Accordingly, to study the role of LncRNA-GSTT1-AS1 in T-cell exhaustion in hematologic malignancies, we evaluated its expression level in CD8^+^ T cells from ALL and AML patients. However, our results showed that the expression of LncRNA-GSTT1-AS1 was not significantly different in CD8^+^ T cells from ALL and AML patients compared to those from control subjects. Based on the results, *SAP* expression was significantly lower in AML patients than in control subjects, while *CD244* and *EAT-2* did not show significant differences between the patients and controls. This finding might be because only six AML patients were enrolled in our study. This is a limitation for this study, which suggests future studies enrolling larger numbers of AML patients.

In conclusion, this study demonstrated a low *SAP*/*CD244* expression ratio in CD8^+^ T cells in ALL patients. Therefore, it can be assumed that CD244 signaling plays an inhibitory role in CD8^+^ T cells in ALL patients, which is in line with T-cell exhaustion in ALL. Further studies are required to perform functional assays on CD8^+^ T cells to elucidate the exact role of CD244 in acute leukemia.

## DECLARATIONS

### Acknowledgments

The authors thank the patients and their families for their support, cooperation, and patience. We would like to thank the staff of the departments associated with care and management of the patients. 

### Ethical statement

All the patients and healthy individuals signed the consent form, which was based on the rules of the Ethics Committee of Mazandaran University of Medical Sciences, Sari, Iran (ethical code: IR.MAZUMS. IMAMHOSPITAL.REC.1399.8204).

### Data availability

The raw data supporting the conclusions of this article are available from the corresponding author upon reasonable request. 

### Author contributions

MM: carried out the assays and contributed to data collection and analysis, and prepared the manuscript; HAO: designed and conducted the research; AN and RV: helped in PCR optimization and analysis of PCR data; HK, EZ, and MT: provided the samples; MT: designed and conducted the research and prepared the manuscript. All authors read and approved the final version of manuscript.

### Conflict of interest

None declared.

### Funding/support

This study was financially supported by Mazandaran University of Medical Sciences, Sari, Iran (grant no.: 8204).
